# Conjunctival dysbiosis in mucosa-associated lymphoid tissue lymphoma

**DOI:** 10.1038/s41598-019-44861-5

**Published:** 2019-06-10

**Authors:** Kazunobu Asao, Noriyasu Hashida, Satoru Ando, Daisuke Motooka, Hiroyuki Kurakami, Shota Nakamura, Daisuke Yamashita, Kazuichi Maruyama, Satoshi Kawasaki, Tomomi Yamada, Tetsuya Iida, Kohji Nishida

**Affiliations:** 1 Department of Ophthalmology, Osaka University Graduate School of Medicine, Osaka University, Osaka, Japan; 2 Department of Ocular Immunology and Regenerative Medicine, Osaka University Graduate School of Medicine, Osaka University, Osaka, Japan; 3 Ako Research Institute, Otsuka Pharmaceutical Co., Ltd., Ako, Japan; 4 Department of Infection Metagenomics, Research Institute for Microbial Disease, Osaka University, Osaka, Japan; 5 Department of Medical Innovation, Osaka University Hospital, Osaka, Japan

**Keywords:** Bacterial pathogenesis, Bacterial genetics, Pathogens, Infection

## Abstract

To investigate the conjunctival microbiota and the association between the development of conjunctival mucosa-associated lymphoid tissue (MALT) lymphoma and dysbiosis, DNA samples were collected from 25 conjunctival MALT lymphoma patients and 25 healthy controls. To compare the microbiota, samples were collected from the following four body locations: conjunctiva, meibomian gland, periocular skin and hand. Extracted DNA was analyzed by 16S rRNA sequences, and libraries were sequenced on an Illumina MiSeq sequencer. The differences in bacteria were characterized by using principal coordinate analysis of metagenomics data, and the differences in bacterial compositions were evaluated by linear discriminant analysis effect size. The conjunctival microbiota of MALT lymphoma patients was compositionally different from that of healthy controls. For the conjunctival MALT lymphoma patients, alterations in the microbial composition were detected, and a remarkable change was detected at the conjunctiva. Detailed analysis showed that a specific population of the microbiota, the genus *Delftia*, was significantly more abundant in conjunctival MALT lymphoma patients, and the genera *Bacteroides* and *Clostridium* were less abundant in the MALT lymphoma patients. A specific microbiota on the ocular surface in conjunctival MALT lymphoma patients was detected, and dysbiosis may play an important role in the pathophysiology of conjunctival MALT lymphoma.

## Introduction

Conjunctival mucosa-associated lymphoid tissue (MALT) lymphomas are known as localized, low-grade tumors, and extranodal marginal zone B-cell MALT lymphoma is the most common histological subtype^1–5^. The frequency of ocular adnexal lymphoma is estimated to be approximately 8% of extranodal lymphomas^3^. For patients with primary lymphoma, the prognosis of primary conjunctival lymphomas is reported to be good, resulting in long-term survival^3^. Histologically, conjunctival MALT lymphoma has similar characteristics to gastric MALT lymphoma in the stomach, and it is thought to be caused by a chronic inflammatory response^6^. Several studies have reported the detection of *Helicobacter pylori* (*H*. *pylori)* DNA in some cases of conjunctival MALT lymphoma and the association of *C*. *psittaci* in its development, and therefore, these microorganisms were thought to be causative pathogens^7,8^. However, the pathophysiology of conjunctival MALT lymphoma has not been fully investigated.

In the human body, a great number and variety of commensal bacteria populate and regulate the homeostatic balance of the host^9^. The microbiota in the human body, including oral, skin, conjunctival, vaginal and respiratory tract microbes, plays an important role in the maintenance of health and disease development^10^. Dysbiosis, or an abnormality of homeostasis in the microbiota, causes several systemic disorders, such as inflammatory bowel diseases, obesity and cardiovascular diseases^11–13^. Previous reports have shown the relationships between the immune system and the commensal microbiota in host defense and tissue repair^14^. Pathological changes in the microbiota, such as *Staphylococcus epidermidis*, a commensal skin bacterium, has been demonstrated to cause opportunistic infections and result in the development of catheter infection, prosthetic valve endocarditis and endophthalmitis^15–18^. The association of dysbiosis with central nervous disorders, such as autism, multiple sclerosis, anxiety-depressive behaviors and functional gastrointestinal disorders, is reported in clinical practice, suggesting the possibility of microbe-based therapies to treat symptoms^15–18^. Thus, alterations of the residual microbiota lead to pathogenic infections or inflammation in the host and even result in fatal conditions^9–18^.

The ocular surface, a part of the conjunctiva-associated lymphoid tissue (CALT), is continuously exposed to the external environment, such as temperature changes, ultraviolet light and oxidative stress^19^. This stress has been implicated in the development of pterygium, dry eye, corneal dystrophy and Fuch’s endothelial dystrophy^19,20^. It is likely that changes in the microenvironment at the ocular surface lead to alteration of the microbiota, resulting in disease development. We hypothesized that mucosal microbial dysbiosis could contribute to immunological changes in the conjunctival mucosa and might be associated with the development of conjunctival MALT lymphoma. To verify this hypothesis, we investigated the microbial diversity in conjunctival MALT lymphoma and healthy controls and compared four body locations to detect their specific microbiota.

In the current study, we detected differences in the microbiota of the conjunctiva between conjunctival MALT lymphoma patients and healthy controls and discussed the role of the microbiota in the pathogenesis of conjunctival MALT lymphoma.

## Results

A total of 50 persons (25 patients, 25 healthy controls) were enrolled in this study, and samples were collected at four locations (conjunctiva, meibomian gland, periocular skin and hand) and at two time points (baseline and 1 month later). After DNA extraction, the DNA was analyzed by 16S rRNA sequencing, and libraries were sequenced on an Illumina MiSeq sequencer. The relative abundances of bacteria in the four locations were compared using JMP and R software. The detailed microbacterial differences were shown using the LefSe program.

### Clinical data of conjunctival MALT lymphoma patients

The clinical data and background for each patient are shown in Table 1. Of 25 patients, 6 patients had a previous history of chemotherapy, i.e. rituximab, R-THP-COP (rituximab, tetrahydropyranyladriamycin, cyclophosphamide, vincristine, and prednisolone) or R-CHOP (rituximab, adriamycin, cyclophosphamide, vincristine, and prednisolone), 3 patients received radiotherapy, and 5 patients received both. Concerning the sample collections during the treatments, we divided samples into three subgroups before treatment (6 patients), under treatment (14 patients), and after treatment (5 patients). Five patients had gastric lesions; numbers of gastric MALT lymphoma, gastric polyp, gastric ulcer and gastric cancer were 2, 1, 1 and 1, respectively. The mean follow-up period was 50.0 ± 6.2 months (range, 5–93 months). In this study, the biopsy after diagnosis and subsequent histological analyses revealed that the type for all patients was extranodal marginal zone B-cell lymphoma of MALT-type lymphoma. And, all cases were B-cell co-receptor markers (CD20) positive immunologically. Additionally, we performed Kappa light chain restriction analyses on two patients, and confirmed monoclonal proliferation in those two cases.

**Table 1:** Demographic data of conjunctival MALT lymphoma patients.

Age	Sex	Diagnosis	Laterality	Involvements	Treatments	Gastric lesions	Follow-up period (month)
42	F	MALT	unilateral	none	biopsy	none	48
78	F	MALT	bilateral	none	biopsy	none	22
26	F	MALT	bilateral	Orbita	biopsy	none	18
91	F	MALT	bilateral	Lacrimal gland	Rituximab, radiotherapy	none	93
45	F	MALT	bilateral	none	Rituximab	none	20
79	F	MALT	bilateral	none	biopsy	MALT lymphoma	7
73	F	MALT	unilateral	none	Rituximab, radiotherapy	none	23
85	F	MALT	unilateral	none	R-THP-COP	none	63
58	F	MALT	unilateral	none	biopsy	none	74
78	M	MALT	unilateral	none	Rituximab	gastric cancer	86
51	M	MALT	unilateral	none	Radiotherapy	gastric ulcer	57
72	M	MALT	unilateral	Lacrimal gland	Radiotherapy	none	53
74	F	MALT	bilateral	Orbita	R-CHOP, radiotherapy	none	89
78	M	MALT	bilateral	Orbita	Rituximab	gastric polyp	64
52	F	MALT	unilateral	none	biopsy	none	66
50	F	MALT	unilateral	none	biopsy	none	15
63	M	MALT	unilateral	none	R-CHOP, radiotherapy	none	90
61	F	MALT	bilateral	none	Radiotherapy	none	85
57	M	MALT	bilateral	none	R-CHOP, radiotherapy	MALT lymphoma	93
47	F	MALT	unilateral	none	Rituximab	none	48
42	F	MALT	unilateral	none	biopsy	none	7
65	M	MALT	unilateral	none	R-CHOP	none	55
67	F	MALT	unilateral	none	biopsy	none	65
51	F	MALT	unilateral	none	biopsy	none	5
58	F	MALT	unilateral	none	biopsy	none	5

F = female; M = male; MALT = extranodal marginal zone B-cell MALT lymphoma.

R-THP-COP = rituximab, tetrahydropyranyladriamycin, cyclophosphamide, vincristine, and prednisolone.

R-CHOP = rituximab, adriamycin, cyclophosphamide, vincristine, and prednisolone.

### DNA sequencing, data processing and stability

**Figure 1:**
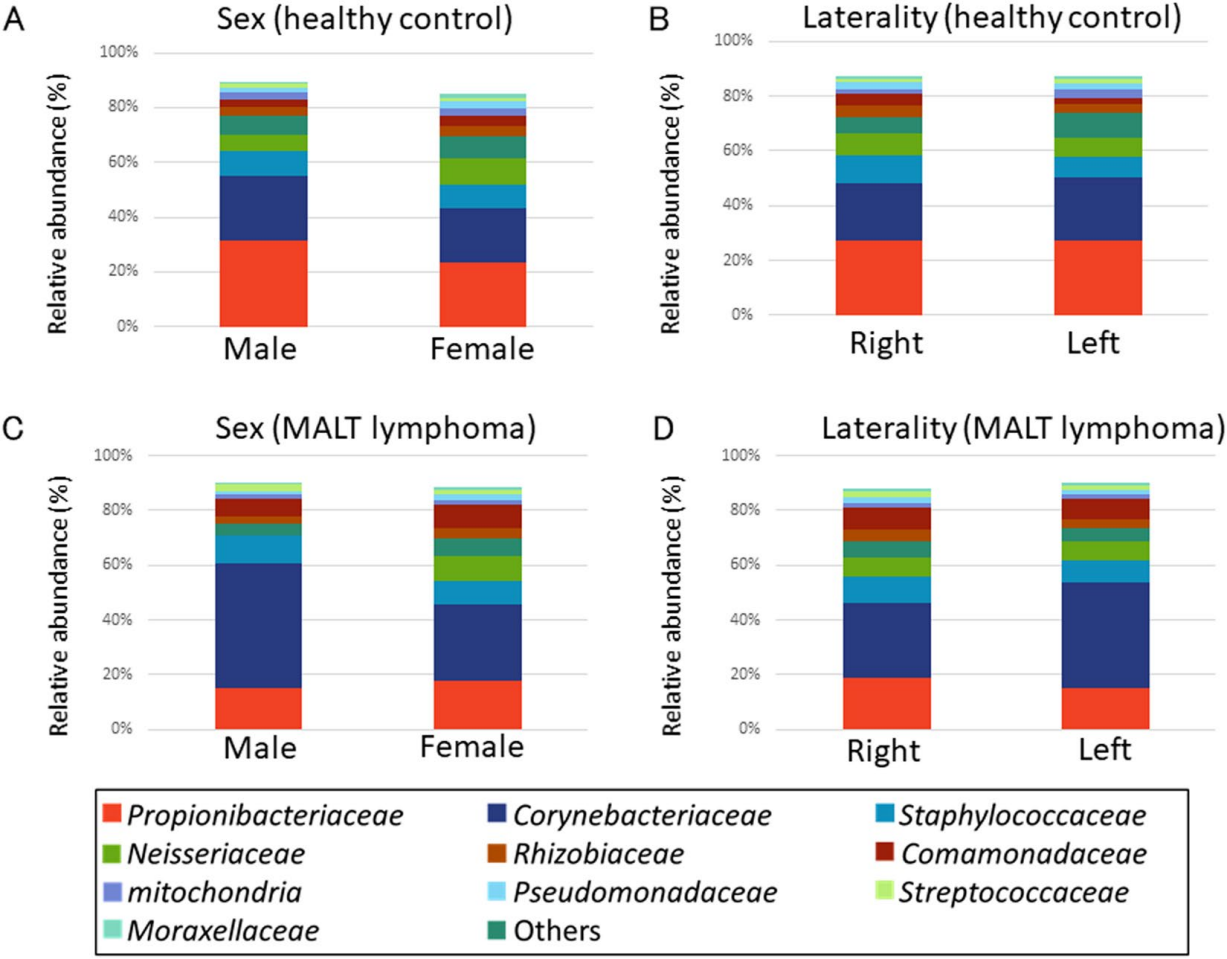
The differences by sex and laterality in the conjunctiva of healthy controls and patients with MALT lymphoma. (**A**,**B**) Relative abundance of top11 compositions in sex and laterality showing that there were no significant variations in the healthy control. (**C**,**D**) For conjunctival MALT lymphoma patients, the differences of sex and laterality on the microbacterial composition of the conjunctiva were not detected. “Others” denotes that this part sums up the microbiota that are not recognized the taxometric classification at the family level.

A total of 18,851,375 raw 16S rRNA gene sequences were obtained, which, after quality filtering, resulted in a total of 13,094,927 paired end sequences with an average of 53,231 sequences per sample. To determine the stability of the microbiota among the four locations, DNA sampling was performed one month after the baseline for healthy controls (Supplemental Fig. 1). There was no significant variation between sampling points regarding the microbacterial composition. The influences of sex and laterality on the microbial compositions of the conjunctiva were evaluated in healthy controls (Fig. 1A,B) and conjunctival MALT lymphoma patients (Fig. 1C,D). There were no significant variations in the groups for laterality or sex.

### Species richness and similarities of microbacterial groups with α diversities

**Figure 2:**
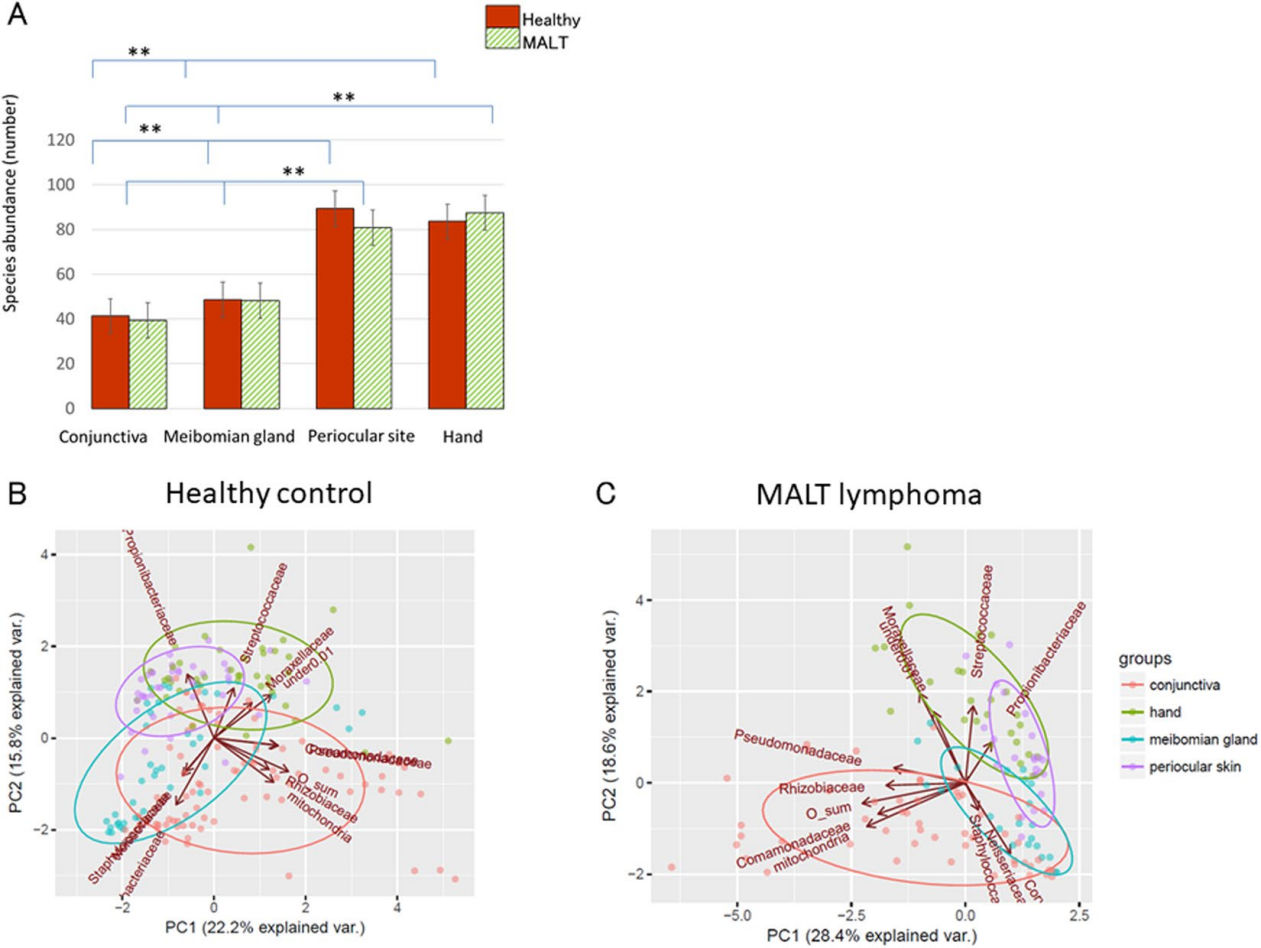
Commensal microbiota at four locations, abundance and principal component analysis (PCA). (**A**) The statistical analyses of microbacterial α diversity at four body locations show that there were no significant differences between the conjunctiva of healthy controls and those of conjunctival MALT lymphoma patients. The conjunctival and meibomian samples show lower α diversity than the periocular skin and hand samples (*P* < 0.01). “**” means significant differences (*P* < 0.05). (**B,C**). The similarities of the bacterial groups in β diversity with PCA showing the distances of the four body locations (conjunctiva (red), meibomian gland (blue), periocular skin (purple) and hand (green)) in healthy controls (**B**) and conjunctival MALT lymphoma patients (**C**).

Microbacterial diversity among the four body locations in patients with conjunctival MALT lymphoma and healthy controls was investigated at the family level (Fig. 2A). The data are presented as the mean ± standard error (SE). The numbers of bacteria in the conjunctiva, meibomian gland, periocular skin, and hand of healthy controls were 41.2 ± 0.86 (range, 33–52), 48.6 ± 2.31 (range, 32–65), 89.4 ± 5.14 (range, 50–131), and 83.5 ± 5.11 (range, 38–137), respectively. The numbers of bacteria in the conjunctiva, meibomian gland, periocular skin, and hand of conjunctival MALT lymphoma patients were 39.4 ± 0.86 (range, 30–51), 48.2 ± 2.48 (range, 31–79), 80.8 ± 6.09 (range, 47–187), and 87.4 ± 6.57 (range, 55–140), respectively. The statistical analysis between healthy controls and conjunctival MALT lymphoma patients was performed using two-way ANOVA with Tukey post hoc. There were no significant differences in bacterial α diversity in each location (conjunctiva: *P* = 1.0, meibomian gland: *P* = 1.0, periocular skin: *P* = 0.12, hand: *P* = 0.93) between healthy controls and conjunctival MALT lymphoma patients. The conjunctival and meibomian samples showed lower α diversities than the periocular skin and hand samples (*P* < 0.01).

The main microorganisms composing the conjunctival microbiota of healthy and MALT lymphoma subjects are shown in Fig. 1. To investigate the similarities in the β diversity among microbiota groups, principal component analysis (PCA) was performed, and four clusters with top 11 compositions were revealed. The distances between the four body locations in healthy controls and conjunctival MALT lymphoma patients are shown in Fig. 2B,C at the family level. There was a relatively small distance between the conjunctiva (red) and the meibomian gland (blue), periocular skin (purple) and hand (green) in healthy controls and conjunctival MALT lymphoma patients. There was a large distance between the conjunctiva and the hand. The separation between the conjunctiva and hand was apparent in the PCA plot.

In addition, we investigated the microbacterial fluctuations provoked by treatments with the permutation analysis of variance (PERMANOVA). However, we could not find any significant differences between before, under, or after treatment.

### The differences of the bacterial compositions between healthy controls and patients with conjunctival MALT lymphoma at the genus level

**Figure 3:**
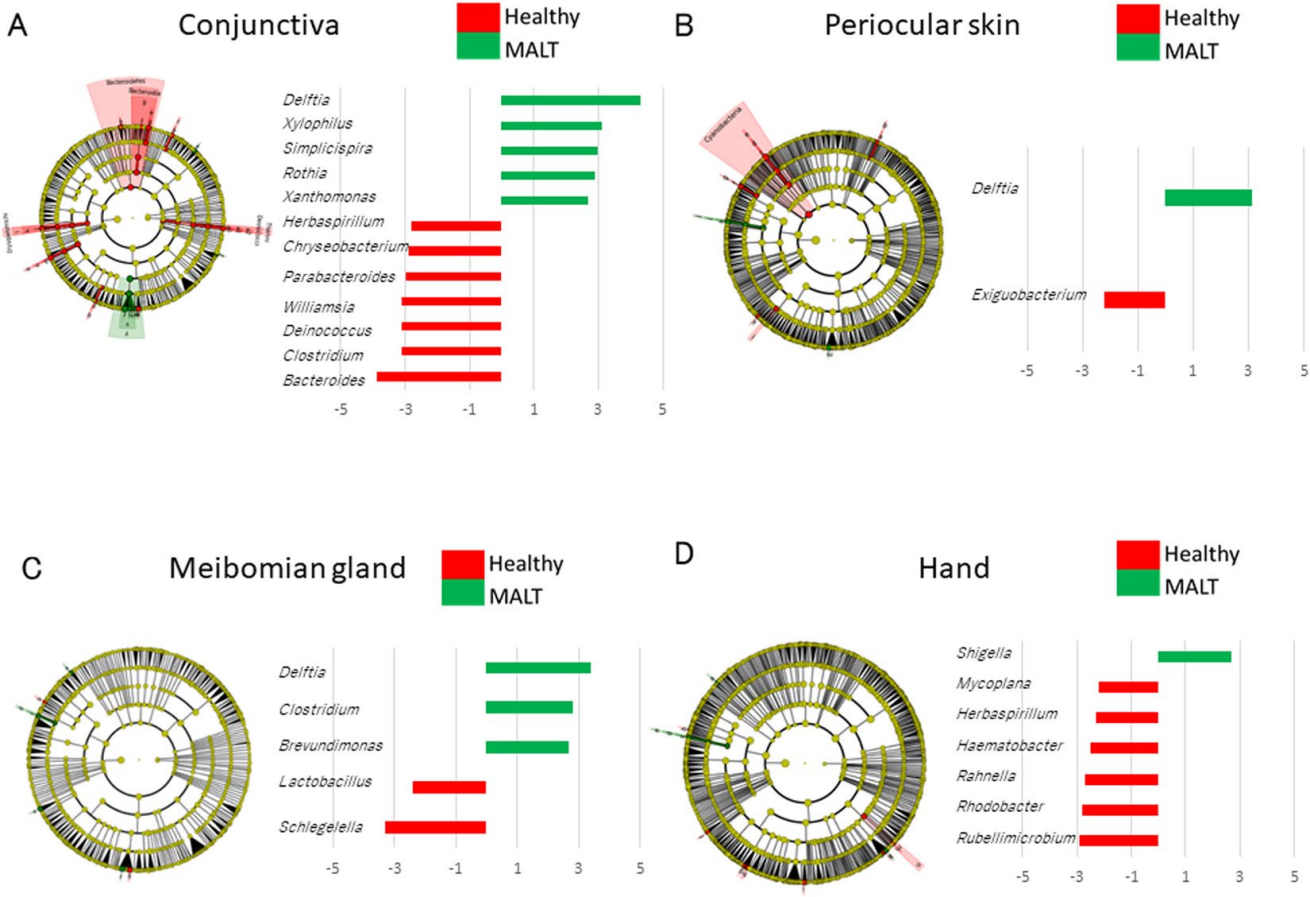
The microbial differences between conjunctival MALT lymphoma patients and healthy controls at four locations analyzed by a linear discriminant analysis effect size program (LEfSe). LefSe data and LDA scores showing a significant increase (green) or decrease (red) at four body locations. A significantly greater abundance of microorganism and a lower abundance of them were observed each in conjunctiva (**A**), periocular skin (**B**), meibomian gland (**C**), and hand (**D**), respectively.

The microorganisms that were significantly increased (green) or decreased (red) in the conjunctival MALT lymphoma subjects were found among the four body locations by an algorism called LefSe (linear discriminant analysis effect size)^21^ with the data classified by genus level. These linear discriminant analysis (LDA) scores are plotted in Fig. 3. A significantly greater abundance of *Delftia*, *Xylophilus*, *Simplicispira*, *Rothia* and *Xanthomonas* and a lower abundance of *Bacteroides*, *Clostridium*, *Deinococcus*, *Williamsia*, *Parabacteroides*, *Chryseobacterium* and *Herbaspirillum* were detected at the conjunctiva in the conjunctival MALT lymphoma group compared to healthy controls (Fig. 3A). On the periocular skin, a significantly greater abundance of *Delftia* and a lower abundance of *Exiguobacterium* were detected (Fig. 3B). At the meibomian gland, a significantly greater abundance of *Delftia*, *Clostridium* and *Brevundimonas* and a lower abundance of *Schlegelella* and *Lactobacillus* were detected (Fig. 3C). On the hand, only *Shigella* was detected in higher abundance and the other microorganisms such as *Mycoplana*, *Herbaspirillum*, *Haematobacter*, *Rahnella*, *Rhodobacter* and *Rubellimicrobium* were lower in abundance in the conjunctival MALT lymphoma patients compared to healthy controls (Fig. 3D).

**Figure 4:**
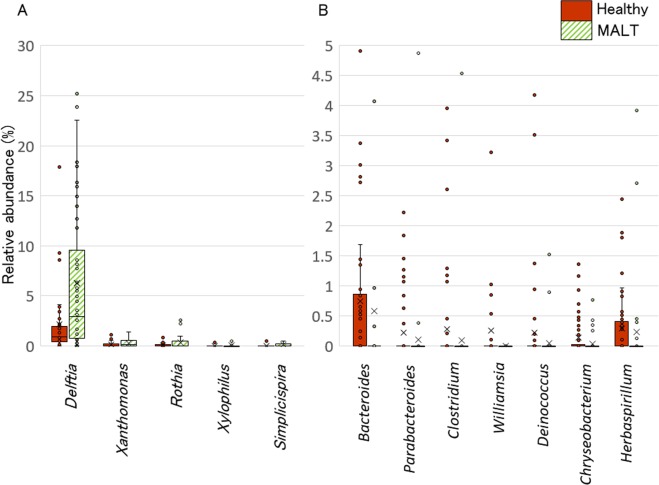
Microbial differences at the conjunctiva between conjunctival MALT lymphoma patients and healthy controls. Box plots showing the bacterial abundance at the conjunctiva between healthy controls (red) and conjunctival MALT lymphoma patients (green). (**A**) *Delftia* was the most prevalent microorganism in MALT lymphoma patients, however; others were detected as significantly prevalent but very rare. (**B)** The prevalence of seven microorganisms were revealed statistically lower in conjunctival MALT lymphoma patients. They were detected mainly in healthy controls and rare in conjunctival MALT cases.

Box plots showed the bacterial prevalence at the conjunctiva in healthy controls and conjunctival MALT lymphoma patients (Fig. 4). The prevalence of *Delftia* was detected in many healthy controls and conjunctival MALT lymphoma patients, and the presence of *Xylophilus*, *Simplicispira*, *Rothia* and *Xanthomonas* was very rare in both groups (Fig. 4A). In contrast, the presence of *Bacteroides*, *Parabacteroides*, *Clostridium*, *Williamsia*, *Deinococcus*, *Chryseobacterium* and *Herbaspirillum* was detected in healthy controls and was rare in conjunctival MALT lymphoma patients (Fig. 4B). The prevalences of seven microorganisms in conjunctival MALT lymphoma were statistically lower than those of healthy controls.

For *Delftia*, *Bacteroides and Clostridium*, in conjunctival MALT lymphoma patients, multivariate analysis was performed on factors such as age, gender, chemotherapy, radiotherapy, laterality, other (orbita and lacrimal gland) involvements (Table 2). There were statistically significant differences for *Delftia* according to other involvements (orbita and lacrimal gland) (*P* = 0.022) and for *Bacteroides* according to chemotherapy (*P* = 0.018). There were no correlations observed in the three microorganisms at the conjunctiva of MALT lymphoma patients and healthy controls.

**Table 2:** Multivariate analysis of three microorganism.

Clinical Parameters	Microorganism	P value
Age (under 60’s vs over 60’s)	*Delftia*	0.6052
	*Bacteroides*	0.8175
	*Clostridium*	0.6022
Gender (male vs female)	*Delftia*	0.9565
	*Bacteroides*	0.0842
	*Clostridium*	0.9744
Laterality (bilateral vs unilateral)	*Delftia*	0.1138
	*Bacteroides*	0.3618
	*Clostridium*	0.3944
Involvement (orbita or lacrimal gland vs no involvement)	*Delftia*	0.0220
	*Bacteroides*	0.7983
	*Clostridium*	0.8189
Chemotherapy (performed vs not performed)	*Delftia*	0.1378
	*Bacteroides*	0.0182
	*Clostridium*	0.2902
Radiotherapy (performed vs not performed)	*Delftia*	0.7386
	*Bacteroides*	0.4528
	*Clostridium*	0.8101
Gastric lesion (positive vs negative)	*Delftia*	0.4624
	*Bacteroides*	0.4020
	*Clostridium*	0.8412

### Physiological changes in the tears of MALT lymphoma patient

**Figure 5:**
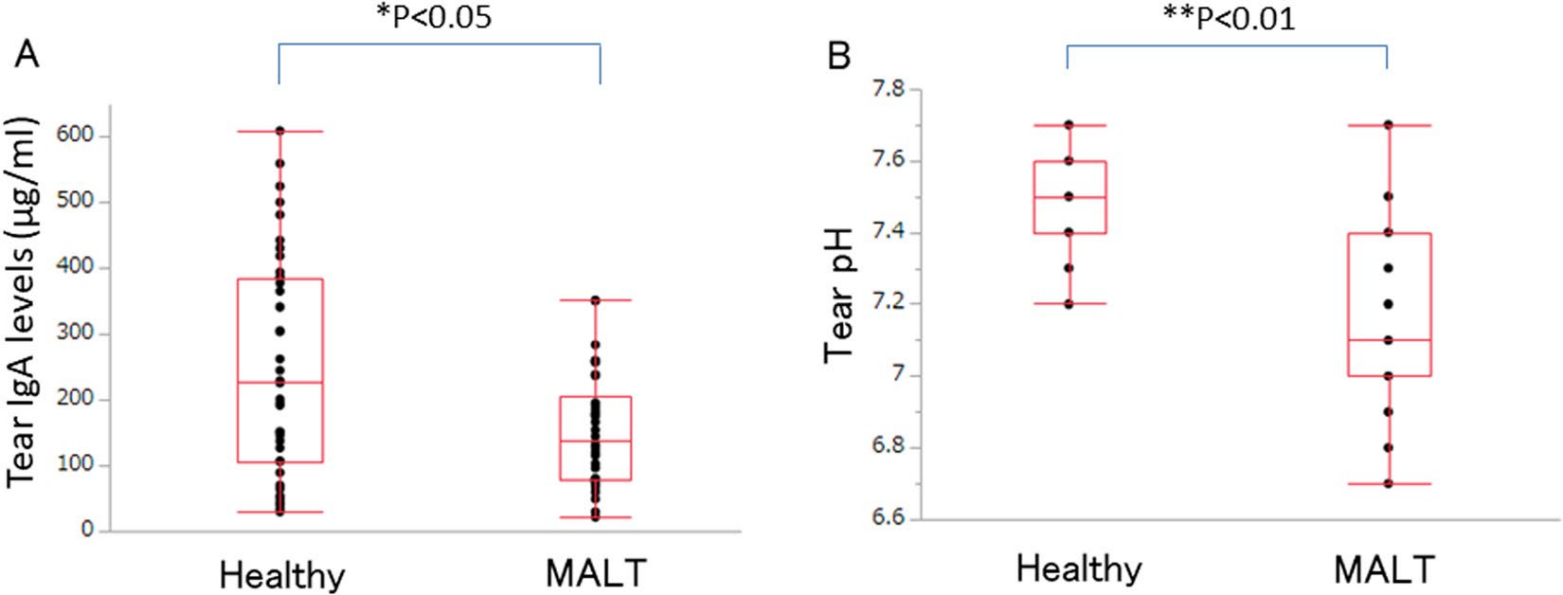
Levels of tear IgA and tear pH in the conjunctiva of healthy controls and conjunctival MALT lymphoma patients. (**A**) There were statistically significant differences of tear IgA level between healthy controls and conjunctival MALT lymphoma patients (Wilcoxon rank sum test, *P* < 0.05). (**B**) There were statistically significant differences of tear pH between healthy controls and conjunctival MALT lymphoma patients (Wilcoxon rank sum test, *P* < 0.01).

Previous reports describe that *Bacteroides* and *Clostridium* maintain ocular surface homeostasis through the production of IgA, which raises a possibility that a decrease of these microorganism could result in lower IgA concentration^22,23^. To this end, we measured the tear IgA levels of MALT lymphoma patients and the healthy controls. The IgA concentration in tears of conjunctival MALT lymphoma patients (153.4 ± 166.9 μg/mL) showed statistically significant difference compared to that of the control group (252.9 ± 87.1 μg/mL) (Wilcoxon rank sum test, *P* < 0.05) (Fig. 5A). On the other hand, the serum IgA concentration from conjunctival MALT lymphoma patients (N = 10) was 206.5 ± 86.6 mg/dL and was within normal limits^24^. A previous report showing that *Delftia* has the ability to utilize glucose oxidatively via catalase and oxidase activity and may change the conjunctival environment^25^, this raises the possibility that on increase of *Delftia* resulted in oxidation of tear pH (normal range, 6.5–7.6)^26^. The tear pH levels of conjunctival MALT lymphoma patients (7.15 ± 0.23) were statistically lower than those of age-matched healthy controls (7.46 ± 0.14) (Wilcoxon rank sum test, *P* < 0.01) (Fig. 5B).

## Discussion

Several studies have shown that the commensal microbiota exists in different body locations^9–13^. In the current study, we showed that the commensal conjunctival microbiota existed against the invasion of foreign microbes, and the conjunctival microbiota of MALT lymphoma patients was compositionally different from that of healthy controls, as highlighted by PCA. A detailed analysis showed that in conjunctival MALT lymphoma patients, the genus *Delftia* was significantly more abundant and the genera *Bacteroides* and *Clostridium* were less abundant than in healthy controls. *Delftia* might have a pathophysiological role in the development of conjunctival MALT lymphoma, and *Bacteroides* and *Clostridium* may have defensive properties in conjunctival MALT lymphoma.

Previous reports showed that a lower abundance of ocular surface microbiota has a great influence on the severity of Sjögren syndrome and dry eye, and the continuous administration of eye drops for glaucoma treatment might affect the microbiota^27–29^. At the meibomian gland, bacteria-related cytotoxicity or inflammation may interfere with the pathological process of meibomian gland dysfunction^30^. Therefore, this altered microbiota might be associated with the development of ocular surface diseases. Previous reports also showed, using PCA, that the skin microbiota is distinct^31^. In each location, the number of conjunctival bacteria was lower than that on the hand and equal to that of the meibomian gland. The conjunctival microbiota differed in composition between conjunctival MALT lymphoma patients and healthy controls. High-diversity skin sites were the palm, finger and foot. On the other hand, the conjunctival microbiota had significantly lower phylogenetic diversity than that of the hand^31^. Some factors, such as humidity, the existence of sebaceous sites, and the use of systemic treatments, have been reported to interfere with conditions^31^. For conjunctival MALT lymphoma, several treatments could be performed to improve the disease prognosis. In this study, no significant difference in the microbial composition among the previous therapies was observed because the dysbiosis of the conjunctival microbiota might already exist during the development of MALT lymphoma, even if some interventions were performed. Environmental characteristics may play an important role in forming the conjunctival bacterial microbiota.

In the human body, mucosal organs, including the conjunctiva, are equipped with characteristic immune defense mechanisms, and the same anatomic mechanism, CALT (which contains MALT), exists at the ocular surface^32,33^. In the stomach, a similar phenomenon could occur in the presence of *H*. *pylori*, which changes the microenvironment of the stomach by changing the urease activity^34^. In our study, the frequency of *Delftia* was significantly higher in the conjunctiva than in the three other body locations for conjunctival MALT lymphoma patients and healthy controls. Previous reports showed that *Delftia* was an aerobic, gram-negative motile bacillus with polar or bipolar flagella^25^. *Delftia* does not produce urease and is catalase and oxidase positive^25^. Characteristically, *Delftia* has the ability to degrade and utilize glucose oxidatively, which may change the conjunctival environment^25^. *Delftia* was isolated from the soil and is known only as a rare pathogen that can affect immunocompromised patients^35^. Clinically, *Delftia* has resistance to antibiotics, such as β-lactams and aminoglycosides, and can adhere to contact lens cases and form biofilms, resulting in the development of both microbial and infiltrative keratitis at the ocular surface^36,37^. The glucose levels in the tears are known to be correlated with the levels in the blood, and several corneal abnormalities, such as healing of the corneal epithelium, are caused by changes in glucose levels^38,39^. *Delftia*, known as rare pathogens, may predispose individuals to the development of conjunctival MALT lymphoma by changing conjunctival conditions to interfere with CALT^25,35^.

In our study, in the conjunctiva of MALT lymphoma, there were two decreased microorganisms, *Bacteroides* and *Clostridium*, which are known as commensals and an anti-allergic microbiota^22^. Previous reports have shown that normal gut bacteria, such as *Bacteroides*, *Bifidobacterium* and *Lactobacillus*, play a protective role in preventing the proliferation of pathogens and form gut-associated lymphoid tissue (GALT) to maintain homeostasis^40^. Similarly, if disruptions of the ocular surface barrier, such as disruptions of CALT and MALT, occur, these changes could trigger ocular inflammation^41^. The development of immune-mediated diseases such as allergies has been hypothesized to arise as a result of deficiencies in exposure to microbial organisms and their products^42^. It is known that *Bacteroides* produces a bacterial polysaccharide, and it directs the cellular and physical maturation of the developing immune system^43^. In the small intestine, *Bacteroides* interacts with dendritic cells at intestinal Peyer’s patches and induces the production and maturation of immunoglobulin A as a protection mechanism in the GALT^23^. Therefore, the existence of *Bacteroides* from birth is essential to maintain the homeostasis of gut immunity, and likewise, bacteria are helpful to develop focal defensive mechanisms at the ocular surface^44^. Several reports suggest that mucosa-associated *Clostridium* populations play an important role in the induction of Tregs and IgA and the suppression of inflammatory and allergic responses^14,23,43–45^. The lowering of tear IgA concentration and pH in conjunctival MALT lymphoma patients suggest that the change in the microbiota profiles impacts the physiological response of the eye. We have not clarified whether the physiological change indeed resulted from specific bacterium or their compositions in this study because of insufficient data size, but there may be a tendency due to treatment especially in the case of *Delftia*. Hence, *Delftia* is thought to play a pathophysiological role in the development of conjunctival MALT lymphoma, and *Bacteroides* and *Clostridium* may play protective roles. The current study raises an intriguing hypothesis that the fluctuation of bacterial compositions causes disturbance of innate immunity in ocular MALT, while further validation is needed to determine whether *Delftia* or other bacteria identified here are responsible for MALT lymphoma.

## Methods

### Study subjects

#### Patients

We studied 25 consecutive patients (50 eyes) (7 men, 18 women; mean age, 61.7 ± 15.6 years) who were diagnosed by biopsy with conjunctival MALT lymphoma and were followed at our hospital between 2015 and 2017. The subtype was extranodal marginal zone B-cell lymphoma of MALT-type lymphoma in all cases. At the same time, 25 age-matched healthy volunteers (50 eyes) (7 men, 18 women; mean age, 58.3 ± 13.0 years) participated in this study as healthy controls. Exclusion criteria included obvious ocular surface disease, history of recent contact lens usage, use of systemic/topical antibiotics or prescription eye medications in the past 12 months, ocular surgery in the last 12 months, active ocular infection, dry eye, systemic diseases such as diabetes, or smoking. The current study adhered to the tenets of the Declaration of Helsinki, the local ethics committee of the Osaka University Medical Hospital approved the study, and written informed consent was obtained from all subjects.

#### Microbacterial sample collection and DNA isolation

Sample collections were performed in a clean ophthalmic treatment room. After instillation of sterile, topical proparacaine, DNA swabs (Osaki Sterilized Cotton Swabs S0475-10, JAPAN) were used to collect samples from the superior and inferior fornixes of the conjunctiva in both eyes. To compare the conjunctiva with other parts of the skin, swabs were taken from the hand, meibomian glands and skin around the eyes. Samples were carefully transferred into DNA LoBind tubes (Eppendorf, Fremont, CA), and all samples were promptly frozen at −80 °C until the time of DNA extraction. DNA was extracted from each sample using a PowerSoil DNA Isolation Kit (MoBio, Carlsbad, CA) according to the manufacturer’s instructions. The extracted genomic DNA was eluted in 100 µl of the kit elution buffer and stored at −20 °C until analysis.

#### Tear sample collections

To investigate the tear immunoglobulin A (IgA) levels, tear samples were collected from 34 eyes of MALT lymphoma patients and 40 eyes of healthy controls without anesthesia. Schirmer test strips (Ayumi Pharmaceutical Co., Tokyo, Japan) were placed at the outer one-third of the temporal lower conjunctival fornix for 5 minutes to collect tears. The strips were stored at −80 °C in glass vials until further analysis. IgA levels were assayed by enzyme-linked immunosorbent assay (E80-102, Bethyl Laboratories, Montgomery, AL, USA). Additionally, the serum from 10 out of 25 patients were collected to measure the IgA levels.

To investigate the tear pH, tears were collected from 30 eyes of MALT lymphoma patients and 26 eyes of healthy controls. Tear fluid were collected from the inferior meniscus of unanesthetized eyes after instillation of 30 μl sterile water by micropipette onto the ocular surface, followed by movement of the eyes to mix the tear fluid content. All the collected tear volumes were over 10 μl and were carefully transferred into DNA LoBind tubes (Eppendorf, Fremont, CA). For the measurement, all samples were measured immediately after sample collection by an ion-selective electrode handheld meter (LAQUA twin B-731; Horiba).

#### 16S rRNA sequencing and data processing

Each library was prepared according to the “Illumina 16S Metagenomic Sequencing Library Preparation Guide” with a primer set (27Fmod: 5′-AGR GTT TGA TCM TGG CTC AG-3′ and 338R: 5ʹ-TGC TGC CTC CCG TAG GAG T-3ʹ) targeting the V1–V2 region of the 1S rRNA gene. Then, 251 bp paired end sequencing of the amplicon was performed on a MiSeq (Illumina) using a MiSeq v2 500 cycle kit. Paired end sequences were merged using PEAR (http://sco.h-its.org/exelixis/web/software/pear/). Merged reads were quality-trimmed with BBtrim (bbmap.sourceforge.net). Twenty thousand reads per sample were randomly selected using random_sequence_sample.pl (ualberta.ca/~stothard/software.html) for further analysis. The processed sequences were clustered into OTUs defined by a 97% similarity cutoff using UCLUST version 1.2.22q. Representative sequences for each OTU were then classified taxonomically by using RDP Classifier version 2.2 and the Greengenes 13_8 database. The bioinformatics pipeline QIIME, version 1.9.1, was used as the informatics environment for all relevant processing of raw sequencing data.

### Statistical analysis

Data are shown as the mean ± SE. Statistical analyses were carried out using JMP software version 9.0 (SAS Inc, Cary, North Carolina, USA) and the R software environment (in the public domain, http://cran.r-project.org/), version 3.1.3. To discover different features of the microbiota in healthy and MALT subjects, the classified data were analyzed by linear discriminant analysis effect size (LefSe). A *P* < 0.05 was considered statistically significant.

## Supplementary information

Stability of bacterial compositions in four locations of healthy controls.
