# Supplementary material for: Conjunctival dysbiosis in mucosa-associated lymphoid tissue lymphoma
Source: Sci Rep. 2019 Jun 10;9:8424. doi: 10.1038/s41598-019-44861-5 (PMC6557838; doi:10.1038/s41598-019-44861-5)
Supplement: Supplementary file 1 — Stability of bacterial compositions in four locations of healthy controls. [file 41598_2019_44861_MOESM1_ESM.pdf]

Supplementary Information

Conjunctival dysbiosis in mucosa-associated lymphoid tissue lymphoma

Kazunobu Asao, Noriyasu Hashida, Satoru Ando, Daisuke Motooka, Hiroyuki Kurakami, Shota Nakamura, Daisuke Yamashita, Kazuichi Maruyama, Satoshi Kawasaki, Tomomi Yamada, Tetsuya Iida and Kohji Nishida

Supplemental Figure. 1 Stability of bacterial compositions in four locations of healthy controls.

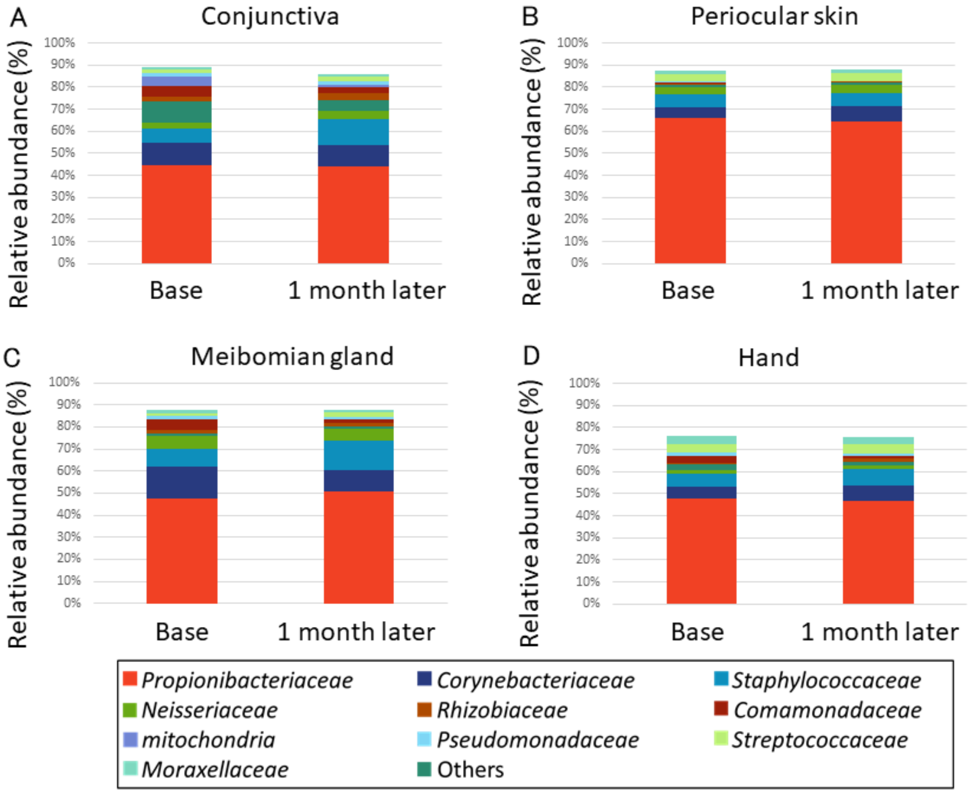

Figure legend

Time course analysis of relative abundance in four locations, conjunctiva (A), periocular skin (B), meibomian gland (C) and hand (D) showed that these compositions did not differ statistically at the time of first sampling (Base) and at one month later in the healthy controls. On the other hand, among four locations, the microbacterial composition of the conjunctiva differed from that of periocular skin and was similar to that of meibomian gland.
